# Exploring the protective association between COVID-19 infection and laryngeal cancer: insights from a Mendelian randomization study

**DOI:** 10.3389/fimmu.2024.1380982

**Published:** 2024-06-10

**Authors:** Heng Wang, Ning Fang, Prithweeraj Mozumder, Richeng Jiang, Xin Wang

**Affiliations:** Department of Otolaryngology Head and Neck Surgery, The First Hospital of Jilin University, Changchun, China

**Keywords:** COVID-19, laryngeal cancer, Mendelian randomization, IFNAR2, immune response, viral infections

## Abstract

**Introduction:**

Viral infections have been implicated as a risk factor for laryngeal cancer. Given the possible effects of Corona virus disease 2019 (COVID-19) on the laryngeal tissue, we investigated the causal link between COVID-19 and laryngeal cancer using a two-sample Mendelian randomization (MR) approach.

**Methods:**

We utilized genetic data from the 5th Genome-wide association studies (GWAS) edition of the COVID-19 Host Genetics Initiative (published on January 18, 2021) and a large-scale laryngeal cancer GWAS comprising 180 cases and 218,612 controls of European ancestry. We applied inverse variance weighting, MR Egger, and weighted median methods to infer causality. We performed sensitivity analysis using the “leave-one-out” method to verify robustness.

**Results:**

We found no evidence of a causal association between gene-predicted COVID-19 and laryngeal cancer [Odds ratio (OR)=0.24 (95% Confidence intervals (CI), 0.05–1.26), P=0.09]. However, we observed significant inverse associations between gene-predicted COVID-19 hospitalization [OR=0.51 (95% CI, 0.28–0.95), P=0.03] and severe patients [OR=0.62 (95% CI, 0.43–0.90), P=0.01] and laryngeal cancer. Notably, the study detected important genetic variants, such as rs13050728, that modulate the expression of interferon alpha receptor 2 (IFNAR2), indicating possible roles for immune response pathways in both COVID-19 and cancer.

**Discussion:**

This study reveals a potential interaction between COVID-19 severity, genetic factors, and laryngeal cancer, underscoring the importance of investigating the immune response mechanisms in both conditions. These findings contribute to the understanding of the complex interactions between COVID-19 and laryngeal cancer and may guide future research on the role of immune response, particularly involving IFNAR2.

## 1 Introduction

Corona virus disease 2019 (COVID-19), characterized by a range of febrile and respiratory symptoms with potentially lethal risks caused by SARS-CoV-2, has garnered widespread global attention since its initial report. After three years of a global pandemic, COVID-19 has resulted in over 700 million infections, leading to millions of deaths (1–3). While the vast majority of COVID-19 patients have recovered, some individuals still experience Post-COVID-19 symptoms, indicating its long-term effects on various organ functions (4).

Laryngeal cancer primarily manifests as squamous cell carcinoma within the larynx. The global annual incidence rate stands at 2.76 per 100,000 inhabitants, with higher prevalence among men than women. Its causes are linked to smoking, alcohol consumption, exposure to physical and chemical stimuli, and variations in hormone secretion levels (5, 6). As a malignant tumor localized in the larynx, laryngeal cancer obstructs patients’ upper respiratory tracts and esophagus, leading to difficulties in breathing and swallowing. Simultaneously, it affects patients’ voices, resulting in hoarseness and articulation challenges (7). Additionally, viral infections, particularly human papilloma virus (HPV), constitute significant risk factors for laryngeal cancer. Studies suggest an approximate 28% HPV infection rate among patients with laryngeal cancer. HPV, upon infecting the larynx, may induce gene mutations and disruptions in gene expression through various mechanisms, thereby causing cell cycle dysregulation, abnormal proliferation, and ultimately leading to the development of laryngeal cancer (8). Furthermore, several studies indicate that HPV-positive patients exhibit a more favorable prognosis than HPV-negative patients, underscoring the role of viral infections in oropharyngeal cancer development and treatment (9–11).

Given that COVID-19 is a viral infection closely associated with the respiratory system, there is curiosity regarding its potential impact on the occurrence of laryngeal cancer through analogous effects. Several case reports have indicated that COVID-19 can prompt acute laryngitis and epiglottitis, hinting at potential adverse effects on laryngeal function (12–14). Previous studies have also highlighted a notable correlation between COVID-19 and the incidence of diverse cancers (15, 16). This has prompted investigations into the potential correlation between COVID-19 and laryngeal cancer. However, laryngeal cancer is a condition with a multifaceted etiology, and conventional randomized controlled studies are susceptible to confounding variables and reverse causation. These studies are also time-consuming and resource-intensive. Hence, this study opts for Mendelian randomization (MR), leveraging genetic variation as an instrumental variable based on Mendel’s law of inheritance. MR helps circumvent confounding factors and reverse causation, allowing for the investigation of the relationship between COVID-19 and laryngeal cancer (17).

## 2 Materials and methods

### 2.1 Study design

**Figure 1:**
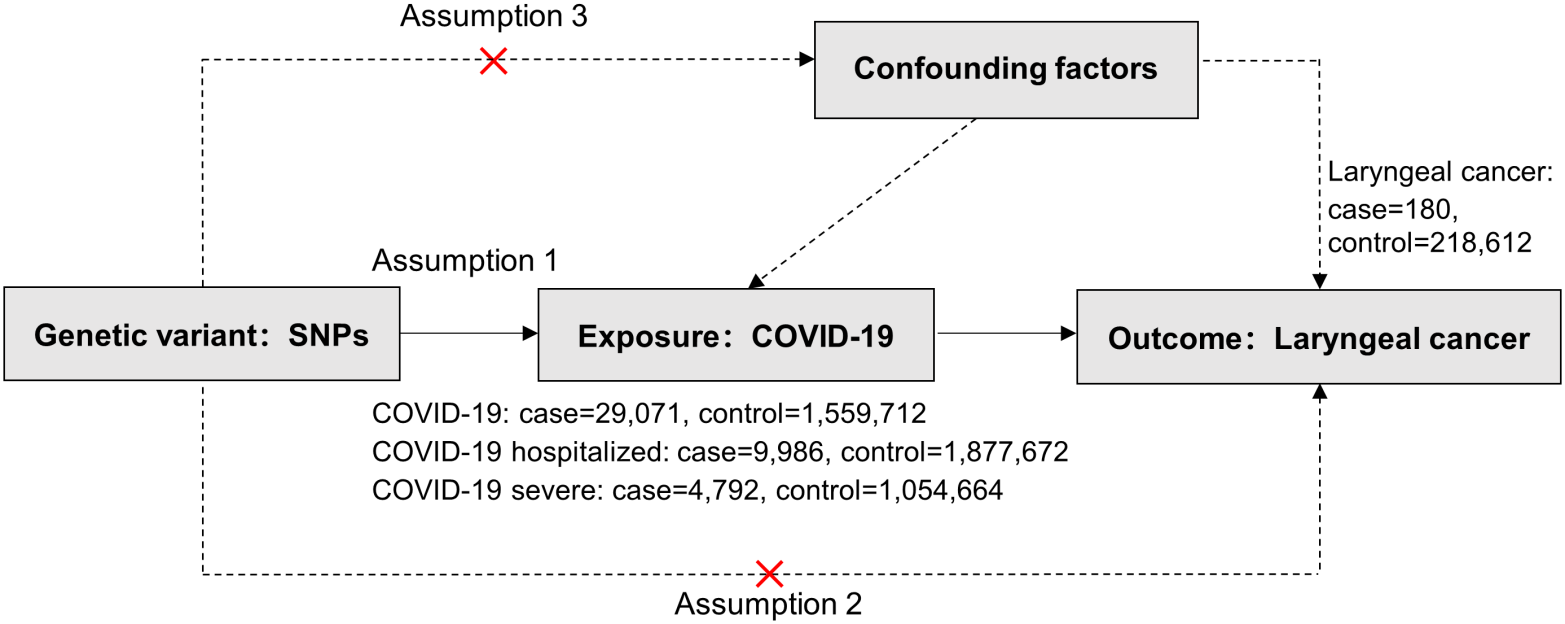
Three critical assumptions of COVID-19 on Laryngeal cancer via Mendelian randomization. Three distinct assumptions are illustrated by three paths. Assumption 1: The association of SNPs with COVID-19 (the exposure). Assumption 2: The SNPs influence laryngeal cancer solely through COVID-19 (as the exposure) and not through any alternative causal pathways. Assumption 3: The SNPs are entirely independent of potential confounding factors that might influence both COVID-19 and laryngeal cancer.

In this study, COVID-19 served as the exposure factor and was categorized based on severity: non-hospitalized patients, hospitalized patients, and those with severe respiratory symptoms. Laryngeal cancer was selected as the outcome factor and investigated using the MR methodology. MR studies hinge on three critical assumptions: association, independence, and exclusivity (18). Our study assumes: (i) a significant association between the instrumental variables (IV, *i.e.*, Single Nucleotide Polymorphism, SNPs) and COVID-19 patients; (ii) independence of SNPs from all confounders of COVID-19 and laryngeal cancer; (iii) the instrumental variables solely impacting laryngeal cancer through their associations with COVID-19 (**Figure 1**).

### 2.2 COVID-19 data sources

Genetic association summary statistics for COVID-19 risk were derived from COVID-19 Genome-wide association studies (GWAS) version 5, released on January 18, 2021, per the 2020 COVID-19 Host Genetics Initiative (19). This database included three phenotypes to explore correlations: non-hospitalized (29,071 patients, 1,559,712 healthy controls), hospitalized (9,986 patients, 1,877,672 healthy controls), and severe respiratory symptom patients (4,792 patients, 1,054,664 healthy controls) from European populations (**Supplementary Table S1**). Ethical approvals were obtained from the original study.

### 2.3 Laryngeal cancer data sources

The laryngeal cancer GWAS dataset was sourced from the FinnGen Biobank’s 5th round of data collection in Finland (20), encompassing 180 patients and 218,612 healthy control European subjects (**Supplementary Table S1**). Ethical approvals were obtained from the original study.

### 2.4 Instrumental variables

Significant SNPs (P<5×10^-8^) related to COVID-19 from the entire genome of 1,000 individuals were compiled. The R^2^ threshold was set to 0.001, and kilobase pairs to 10,000 to eliminate linkage disequilibrium interference. Heterogeneity tests removed significantly divergent SNPs, yielding valid SNPs associated with COVID-19 as instrumental variables. An F value >10 indicated the absence of weak instrumental variable bias ( $\text{F}=\frac{N-K-1}{K}\times \frac{R}{1+R}$ , where N is the sample size, K is the number of SNPs, and R^2^ is the variance proportion explained by the SNPs) (21). R^2^ was calculated as ${\text{R}}^{2}=\frac{2\times \text{EAF}\times \left(1-\text{EAF}\right)\times \beta^{2}}{{\text{SD}}^{2}}$ , where EAF represents the frequency of the effect allele, β denotes the allele effect value, and SD stands for the standard deviation (22).

### 2.5 Mendelian randomization analysis

MR analysis, an effective epidemiological tool, mitigates confounding factors and reverse causality. This study primarily employed inverse variance-weighted (IVW), MR-Egger regression, and weighted median methods for analysis. Utilizing these approaches, the causal impact of the exposure on the outcome was computed by determining the ratio of the SNP associated with the exposures (Wald ratio estimate). IVW weighs each instrumental variable’s inverse variance, providing the standard MR analysis result (23). MR-Egger considers the intercept term in regression and uses inverse variance as weights (24). Weighted median estimator (WME) yields consistent causality estimates if at least half of the valid instruments are available (25). IVW results hold more significance than MR-Egger and WME methods. All analyses were carried out utilizing R version 4.3.1, and the MR analysis was performed using version 0.5.7 of the “TwoSampleMR” package.

### 2.6 Sensitivity analysis

The heterogeneity test assessed differences between individual IVs, highlighting significant differences as considerable heterogeneity. The pleiotropy test evaluated horizontal pleiotropy in multiple IVs, primarily using MR-Egger’s intercept term. A non-zero intercept suggests horizontal pleiotropy (26).

This study utilized the “Leave-one-out” method to assess result reliability. Eliminating each IV in turn and comparing the resulting total against the initial MR result determined whether an IV had a non-specific effect on estimation results (27).

## 3 Results

**Figure 2:**
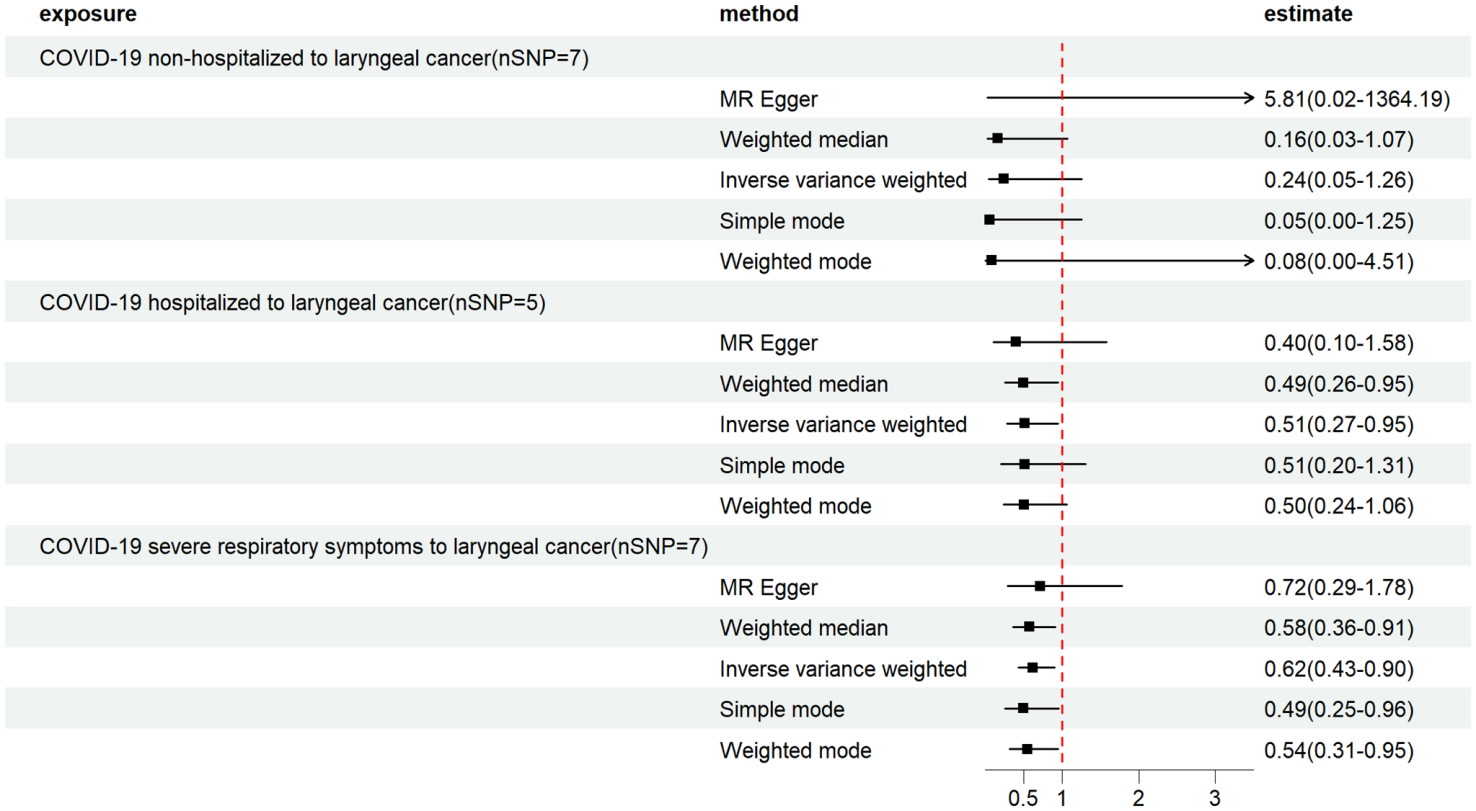
Mendelian randomization estimates from instrument variants for COVID-19 on laryngeal cancer. Depicts the causal relationships between COVID-19, hospitalized COVID-19 and severe COVID-19, and laryngeal cancer through the utilization of five distinct MR methods.

**Figure 3:**
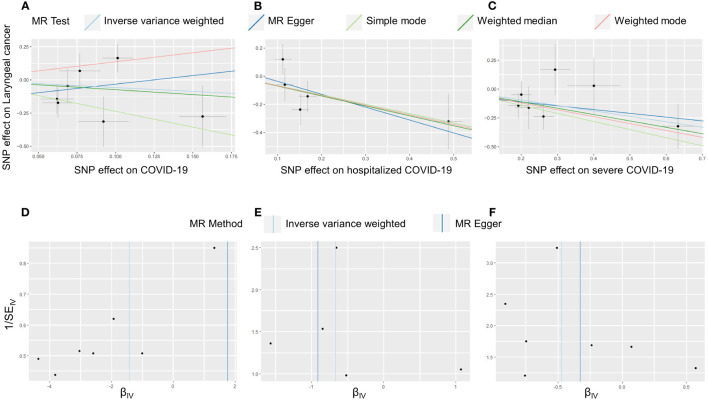
Causal effect of COVID-19 on laryngeal cancer. Scatter plots and funnel plots of Mendelian randomization analyses for the effect of COVID-19 **(A, D)**, hospitalized COVID-19 **(B, E)** and severe COVID-19 **(C, F)** on laryngeal cancer. **(A–C)** The association between the SNP effect size estimate for COVID-19 (on the x-axis) and the corresponding effect size estimate for laryngeal cancer outcomes (on the y-axis) is illustrated. The x-axis trait signifies the exposure, the y-axis trait signifies the outcome, and each intersection point denotes an instrumental variant. The dots and brief lines traversing the dots indicate the effect sizes **(B)** and the 95% confidence intervals (CI) of COVID-19 concerning the outcomes of laryngeal cancer. **(D–F)** The funnel plots show the inverse variance weighted MR estimate of COVID-19 with Laryngeal cancer versus 1/standard error.

In the present study, MR analyses of COVID-19 and laryngeal cancer were conducted using primarily three methods: MR-Egger, IVW, and WME. COVID-19 patients were categorized into three phenotypes based on severity: non-hospitalized, inpatients, and those with severe respiratory symptoms. **Figure 2** illustrates the effect estimates of the MR analyses regarding the association between COVID-19 and laryngeal cancer. We utilized IVW, MR-Egger, simple mode, weighted median, and weighted mode-based estimations to assess the causal associations between genetically predicted COVID-19 severity and laryngeal cancer (**Figure 3**).

For the non-hospitalized COVID-19 patient group, 7 SNP loci were selected as instrumental variables (**Supplementary Table S2**). The IVW method’s results indicated an Odds ratio (OR) of 0.24 (95% Confidence intervals (CI), 0.05–1.26) with a P-value of 0.09, suggesting no correlation between non-hospitalized COVID-19 patients and laryngeal cancer (**Figure 2**; **Table 1**). The Cochran Q test showed no heterogeneity (P=0.13), and the MR-Egger intercept test demonstrated no horizontal pleiotropy (P=0.29) (**Table 1**).

**Table 1:** Causal effect of COVID-19 on the laryngeal cancer.

Exposure	Method	b(se)	OR [95%CI]	N_IV	Q_P	Egger_ intercept	P_ pleiotropy	P
**COVID-19**	MR-Egger	1.76(2.78)	5.81[0.02–1364.19]	7	0.175197	-0.23797	0.285828	0.555
**COVID-19**	IVW	-1.42(0.84)	0.24[0.05–1.26]	7	0.130543	NA	NA	0.092
**COVID-19**	WM	-1.81(0.96)	0.16[0.03–1.07]	7	NA	NA	NA	0.059
**COVID-19 (hospitalized vs population)**	MR-Egger	-0.92(0.70)	0.40[0.10–1.58]	5	0.19632	0.05444	0.70937	0.283
**COVID-19 (hospitalized vs population)**	IVW	-0.67(0.31)	0.51[0.28–0.95]	5	0.29267	NA	NA	0.034*
**COVID-19 (hospitalized vs population)**	WM	-0.71(0.33)	0.49[0.26–0.95]	5	NA	NA	NA	0.034*
**COVID-19 (very severe respiratory confirmed vs population)**	MR-Egger	-0.33(0.46)	0.72[0.29–1.78]	7	0.52363	-0.04745	0.73825	0.510
**COVID-19 (very severe respiratory confirmed vs population)**	IVW	-0.47(0.19)	0.62[0.43–0.90]	7	0.63546	NA	NA	0.013*
**COVID-19 (very severe respiratory confirmed vs population)**	WM	-0.55(0.23)	0.58[0.36–0.91]	7	NA	NA	NA	0.018*

IVW, inverse variance weighted; WM, weighted median; OR, odds ratio; CI, confidence interval; N_IV, number of instrumental variables; Q_P, Cochran’s P-value of heterogeneity analysis. *P< 0.05 was deemed indicative of statistical significance. NA, Not applicable.

In the COVID-19 inpatient group, 5 SNP loci were chosen as instrumental variables (**Supplementary Table S2**). The IVW method revealed an OR of 0.51 (95% CI, 0.28–0.95) with a P-value of 0.03, suggesting an inverse correlation between COVID-19 inpatients and laryngeal cancer (**Figure 2**; **Table 1**). The Cochran Q test showed no heterogeneity (P=0.29), and the MR-Egger intercept test indicated no horizontal multiplicity (P=0.70) (**Table 1**).

Within the COVID-19 patients exhibiting severe respiratory symptoms, 7 SNP loci were utilized as instrumental variables (**Supplementary Table S2**). The IVW method showed an OR of 0.62 (95% CI, 0.43–0.90) with a P-value of 0.01, suggesting a significant inverse correlation between COVID-19 patients with severe respiratory symptoms requiring hospitalization and laryngeal cancer (**Figure 2**; **Table 1**). The Cochran Q test indicated no heterogeneity (P=0.64), and the MR-Egger intercept test revealed no horizontal multiplicity (P=0.74) (**Table 1**).

**Figure 4:**
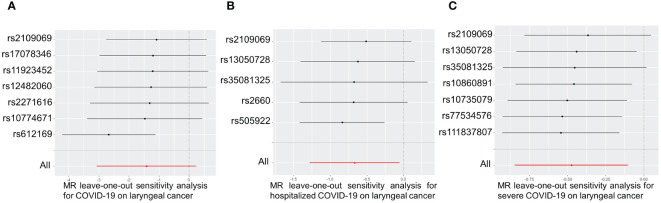
Leave-one-out of SNPs associated between COVID-19 with laryngeal cancer. Leave-one-out of SNPs associated between COVID-19 **(A)**, hospitalized COVID-19 **(B)** and severe COVID-19 **(C)** with laryngeal cancer. Each black point represents the outcome of the Inverse Variance Weighted (IVW) MR method, estimating the causal effect by excluding a specific SNP from the analysis. Each red point represents the IVW estimate using all SNPs.

The causal effect of each SNP associated with COVID-19 and laryngeal cancer was shown in **Supplementary Figure S1**. Leave-one-out sensitivity analysis was performed for each group of COVID-19. Results indicated that after individually removing each SNP, the remaining SNP analysis results closely mirrored the initial analysis, suggesting robust and unbiased data (**Figure 4**).

## 4 Discussion

It has been demonstrated that COVID-19, as a viral disease, can cause acute inflammation of the larynx and epiglottis (12–14). This suggests that SARS-CoV-2 might have the potential to infect the larynx or pharynx similarly to HPV (8). Patients co-infected with HPV and afflicted with laryngeal or oropharyngeal cancer have also exhibited favorable responses to immunotherapy and chemotherapy (10, 11). HPV infection has been suggested as a prognostic indicator for laryngeal and oropharyngeal cancer. Moreover, as a global pandemic, COVID-19 has left many patients with various degrees of post-recovery sequelae, highlighting the potential long-term effects of COVID-19 infection on organ function (4). This has prompted our interest in investigating the correlation between the severity of COVID-19 infection and laryngeal cancer, with the aim of utilizing MR to explore whether COVID-19 infection can serve as a predictive factor for the onset and prognosis of laryngeal cancer. Few MR studies have explored the relationship between the severity of COVID-19 infection and the risk of various cancers, including laryngeal and oropharyngeal cancers (15, 16). However, these studies have not extensively investigated the specific association between COVID-19 and laryngeal cancer. Our research aims to fill this gap by comprehensively examining the correlation between COVID-19 and laryngeal cancer, while also analyzing potential pathways that may influence this relationship.

Despite HPV being a significant risk factor for oropharyngeal cancer, studies consistently show better outcomes among HPV-positive patients, highlighting viral infections’ pivotal role in oropharyngeal cancer’s development and treatment (9–11). Interestingly, we found similar effect of COVID-19 on laryngeal cancer. Our study reveals a significant correlation between both COVID-19 hospitalized patients (OR=0.51, 95% CI: 0.28–0.95, P=0.03) and patients with severe respiratory symptoms (OR=0.62, 95% CI: 0.43–0.90, P=0.01) and laryngeal cancer, indicating a possible protective role against laryngeal cancer. Such results raise the question of why a disease widely impacting the respiratory system could act as a protective factor for laryngeal cancer. Thus, we commenced our investigation with SNP loci associated with COVID-19. Specifically, five SNP loci related to COVID-19 hospitalized patients and seven SNP loci linked to COVID-19 patients with severe respiratory symptoms were chosen as instrumental variables, with three overlapping loci: rs13050728, rs2109069, rs35081325. These three SNPs are believed to elucidate the correlation between COVID-19 and laryngeal cancer.

Previous research (28) has highlighted the strong connection between a genetic marker, rs13050728, and severe COVID-19 cases, influencing the expression of a key immune gene called interferon alpha receptor 2 (IFNAR2). This gene’s role in both COVID-19 and cancer is being explored in this study. Specifically, the C allele of rs13050728, identified in this research, demonstrates a robust link with heightened IFNAR2 expression, potentially impacting immune response mechanisms (28).

Type I interferons (IFNs), pivotal in combating infections and tumors, have been extensively studied (29–32). In the context of COVID-19, delays in the body’s IFN response have been observed, leading to more severe symptoms in affected individuals (28, 33–36). Interestingly, individuals with severe COVID-19 cases and certain cancer patients display escalated levels of IFNAR2 (37, 38). Multiple studies have also confirmed that high expression of IFNAR2 has a significant impact on the good prognosis of immunotherapy, and has a positive impact on patient survival rate and survival time (39–41). This study underscores IFNAR2’s vital role in immune responses to infections and tumors in both diseases. We propose that interference with IFN α and IFN β hinders type I interferon response, impacting COVID-19 severity and increasing IFNAR2 expression. Maintaining high expression of IFNAR2 and restoring normal levels of type I interferon can effectively inhibit the proliferation of tumor cells, thereby reducing the risk of laryngeal cancer or improving the treatment effect of laryngeal cancer patients during IFN treatment.

Laryngeal cancer, often linked to viral infection, paradoxically exhibits improved prognoses in viral-infection-positive cases. A study assessing anti-PD-1 treatment in nasopharyngeal carcinoma patients who received the COVID-19 vaccine reported higher objective response and disease control rates than the non-vaccinated subgroup (42). Meanwhile, certain related studies have suggested that COVID-19 vaccine administration could enhance the therapeutic efficacy of PD-1 inhibitors in various cancers (43, 44). These studies also suggest that COVID-19 vaccination leads to an increase in CD4+ T cell levels in patients, potentially aiding in the activation of CD4+ T cells and reshaping the tumor microenvironment (43). HPV-positive laryngeal cancer patients display extended survival periods and enhanced sensitivity to treatments like immunotherapy and chemotherapy (45–48). This suggests viral infections both pose a risk for laryngeal cancer and positively impact its prognosis, aligning with our findings. We hypothesize that viral infections activate the immune system, potentially influencing a better prognosis.

Use of MR in our study, employing a European population and the largest COVID-19 GWAS, ensured robustness and minimized confounding factors (19). We validated our selected SNPs as instrumental variables, observing no significant heterogeneity or horizontal pleiotropy. However, limitations exist: (i) the novelty of the COVID-19 outbreak might not reflect short-term outcomes in laryngeal cancer, and high mortality rates early in the pandemic may introduce bias; (ii) while statistical methods addressed current issues, alternative pathways influenced by instrumental variables in laryngeal cancer cannot be entirely ruled out; (iii) due to the limited availability of publicly accessible GWAS data on COVID-19 patients and laryngeal cancer, it is currently not feasible to assess the impact of gender and age on their correlation. Further investigation into COVID-19-related genes and their impact on patients is crucial.

In summary, our research suggests that the exacerbation of COVID-19 infection may be an important factor in reducing the risk of laryngeal cancer, and the increase in IFNAR2 levels may play a significant role in this. This inference underscores the significant role of the type I interferon response in mitigating the risk of laryngeal cancer, thereby suggesting that interferon-related immunotherapy holds substantial promise for both the prevention and treatment of this condition. Furthermore, such immunotherapy interventions may offer considerable potential for reducing tumor staging preoperatively and prolonging survival postoperatively among patients with laryngeal cancer.

## 5 Conclusions

In conclusion, genes associated with COVID-19 development might hinder tumor development, potentially lowering laryngeal cancer risk or improving prognoses in severe COVID-19 cases post-immunotherapy. Detailed mechanisms warrant exploration through additional studies.

## Data availability statement

The original contributions presented in the study are included in the article/**Supplementary Material**. Further inquiries can be directed to the corresponding author/s.

## Ethics statement

Due to the utilization of published studies and publicly available databases, no additional ethical approval from an institutional review board was necessary.

## Author contributions

HW: Data curation, Formal analysis, Methodology, Validation, Writing – original draft, Writing – review & editing. NF: Data curation, Methodology, Supervision, Validation, Writing – review & editing. PM: Writing – original draft, Writing – review & editing. RJ: Conceptualization, Data curation, Formal analysis, Investigation, Methodology, Project administration, Resources, Supervision, Validation, Visualization, Writing – original draft, Writing – review & editing. XW: Funding acquisition, Supervision, Writing – original draft, Writing – review & editing.
