# Supplementary material for: Exploring the protective association between COVID-19 infection and laryngeal cancer: insights from a Mendelian randomization study
Source: Front Immunol. 2024 Jun 10;15:1380982. doi: 10.3389/fimmu.2024.1380982 (PMC11194353; doi:10.3389/fimmu.2024.1380982)
Supplement: Supplementary file 1 [file DataSheet_1.docx]

Supplementary Material

##
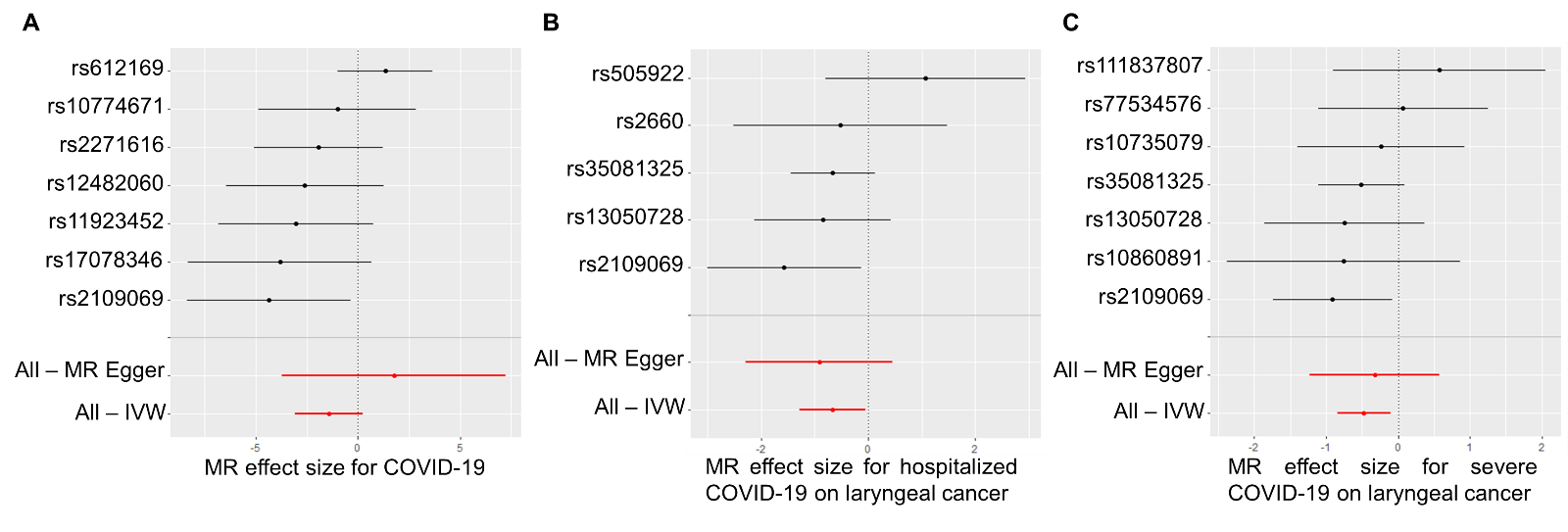


**Supplementary Figure S1. Forest plot of Wald ratio estimate for each SNP associated between COVID-19 with laryngeal cancer.** Each black horizontal line represents Wald ratio estimate of each SNP associated between COVID-19 (A), hospitalized COVID-19 (B) and severe COVID-19 (C) with laryngeal cancer. The solid red line positioned entirely on the left signifies a negative correlation, while its complete presence on the right indicates a positive correlation. The solid line crossing the 0 baseline indicates a non-significant result. IVW: Inverse Variance Weighted.

| Trait | Year | GWAS ID | ncase | ncontrol | nsnp | Population | PMID |
| --- | --- | --- | --- | --- | --- | --- | --- |
| COVID-19 | 2020 | ebi-a-GCST011071 | 29,071 | 1,559,712 | 8,103,014 | European | 32404885 |
| COVID-19 (hospitalized vs population) | 2020 | ebi-a-GCST011081 | 9,986 | 1,877,672 | 8,107,040 | European | 32404885 |
| COVID-19 (very severe respiratory confirmed vs population) | 2020 | ebi-a-GCST011077 | 4,792 | 1,054,664 | 7,496,658 | European | 32404885 |
| Malignant neoplasm of larynx | 2021 | finn-b-C3_LARYNX | 180 | 218,612 | 16,380,466 | European | NA |

**Supplementary Table S1. COVID-19 and laryngeal cancer genome-wide association study (GWAS).** COVID-19: corona virus disease 2019; GWAS: genome-wide association study; GWAS ID: GWAS identity; ncase: the number of COVID-19 case or laryngeal cancer case; ncontrol: the number of the control; nsnp: the number of single-nucleotide polymorphism; PMID, pubMed unique identifier

| SNP | | Position | CHR | EAF | EA | OA | β | SE | P | R^2^ |  |
| --- | --- | --- | --- | --- | --- | --- | --- | --- | --- | --- | --- |
|  | COVID-19 (RELEASE 5) | | | | | | | | | | |
| rs17078346 | | 45845748 | 3 | 0.1055 | C | A | 0.082507 | 0.014033 | 4.11E-09 | 2.18E-05 |  |
| rs11923452 | | 101433515 | 3 | 0.3517 | C | T | -0.05678 | 0.0090576 | 3.64E-10 | 2.47E-05 |  |
| rs2271616 | | 45838013 | 3 | 0.1171 | T | G | 0.14213 | 0.014149 | 9.63E-24 | 6.35E-05 |  |
| rs612169 | | 136143442 | 9 | 0.3529 | G | A | 0.09055 | 0.0090561 | 1.54E-23 | 6.29E-05 |  |
| rs10774671 | | 113357193 | 12 | 0.6719 | A | G | 0.060195 | 0.0089849 | 2.09E-11 | 2.82E-05 |  |
| rs2109069 | | 4719443 | 19 | 0.3161 | A | G | 0.054424 | 0.0097955 | 2.76E-08 | 1.94E-05 |  |
| rs12482060 | | 34611571 | 21 | 0.3417 | G | C | 0.055526 | 0.0094127 | 3.66E-09 | 2.19E-05 |  |
|  | COVID-19 (hospitalized vs population) RELEASE 5 | | | | | | | | | | |
| rs35081325 | | 45889921 | 3 | 0.08122 | T | A | 0.48825 | 0.031508 | 3.68E-54 | 1.27E-05 |  |
| rs505922 | | 136149229 | 9 | 0.3501 | C | T | 0.11182 | 0.019056 | 4.42E-09 | 1.82E-05 |  |
| rs2660 | | 113357442 | 12 | 0.6902 | A | G | 0.11639 | 0.019406 | 2.00E-09 | 1.91E-05 |  |
| rs2109069 | | 4719443 | 19 | 0.3227 | A | G | 0.15131 | 0.019906 | 2.94E-14 | 3.06E-05 |  |
| rs13050728 | | 34615210 | 21 | 0.6528 | C | T | -0.16832 | 0.020183 | 7.44E-17 | 3.68E-05 |  |
|  | COVID-19 (very severe respiratory confirmed vs population) RELEASE 5 | | | | | | | | | | |
| rs35081325 | | 45889921 | 3 | 0.07998 | T | A | 0.63267 | 0.044434 | 5.29E-46 | 1.91E-04 |  |
| rs111837807 | | 31121232 | 6 | 0.13 | C | T | 0.29364 | 0.040997 | 7.93E-13 | 4.84E-05 |  |
| rs10860891 | | 103014757 | 12 | 0.8666 | A | C | -0.22108 | 0.038305 | 7.85E-09 | 3.14E-05 |  |
| rs10735079 | | 113380008 | 12 | 0.6961 | A | G | 0.20047 | 0.027155 | 1.55E-13 | 5.14E-05 |  |
| rs77534576 | | 47940666 | 17 | 0.04097 | T | C | 0.40059 | 0.072355 | 3.09E-08 | 2.89E-05 |  |
| rs2109069 | | 4719443 | 19 | 0.331 | A | G | 0.26147 | 0.027415 | 1.46E-21 | 8.59E-05 |  |
| rs13050728 | | 34615210 | 21 | 0.6374 | C | T | -0.19225 | 0.027406 | 2.31E-12 | 4.64E-05 |  |

**Supplementary Table S2. Association of COVID-19 genetic instrumental variables (IVs) with laryngeal cancer.** IVs: instrumental variables; COVID-19: corona virus disease 2019; GWAS: genome-wide association study; SNP: single-nucleotide polymorphism; CHR: chromosome; EA: effect allele; OA: other allele; β: the regression coefficient based on pyroglutamine raising effect allele; SE, standard error; R^2^: coefficient of determination.
